# Development of the human heart

**DOI:** 10.1002/ajmg.c.31778

**Published:** 2020-02-12

**Authors:** Marieke F.J. Buijtendijk, Phil Barnett, Maurice J.B. van den Hoff

**Affiliations:** ^1^ Department of Medical Biology AmsterdamUMC location AMC Amsterdam The Netherlands

**Keywords:** cardiac development, valve development, septation, epicardial development, cardiac growth

## Abstract

In 2014, an extensive review discussing the major steps of cardiac development focusing on growth, formation of primary and chamber myocardium and the development of the cardiac electrical system, was published. Molecular genetic lineage analyses have since furthered our insight in the developmental origin of the various component parts of the heart, which currently can be unambiguously identified by their unique molecular phenotype. Moreover, genetic, molecular and cell biological analyses have driven insights into the mechanisms underlying the development of the different cardiac components. Here, we build on our previous review and provide an insight into the molecular mechanistic revelations that have forwarded the field of cardiac development. Despite the enormous advances in our knowledge over the last decade, the development of congenital cardiac malformations remains poorly understood. The challenge for the next decade will be to evaluate the different developmental processes using newly developed molecular genetic techniques to further unveil the gene regulatory networks operational during normal and abnormal cardiac development.

## 1 INTRODUCTION

In 2014, Sylva, van den Hoff, & Moorman, 2014 published an extensive review discussing the major steps of cardiac development focusing on growth, formation of primary and chamber myocardium and the development of the cardiac electrical system. Here, we will build on our previous review and on the latest molecular and cell biological studies that have channeled our insights. In addition, we will address post‐natal cardiogenesis, since it has become evident that cardiac development is not complete at birth and that, contrary to traditional views, the adult heart can no longer be considered a post‐mitotic organ.

In the healthy normal post‐natal heart, oxygen‐rich blood enters the left atrium, is propagated to the left ventricle and then pumped via the aorta into the systemic circulation. The oxygen‐deprived blood, returning from the body, enters the right atrium and is propelled by the right ventricle via the pulmonary trunk toward the lungs. The cardiac conduction system orchestrates the efficient contraction‐relaxation cycle of the atria and ventricles. The electrical impulse resulting in cardiac contraction is triggered in the sinus node, which is located at the entrance of the superior caval vein into the right atrium. The electrical impulse spreads through both atria, but cannot directly activate the ventricles due to the electrical isolation of the atria from the ventricles by the annulus fibrosus (also called insulating plane or fibrous continuity). The electrical impulse is delayed in the atrioventricular (AV) node, and then quickly propagated through the His‐bundle (AV‐bundle), which penetrates the insulating annulus fibrosis plane, via the bundle branches and the peripheral conduction system (the Purkinje fibers) to the cardiomyocytes. The coordinated propagation of the electrical impulse ensures the synchronous contraction of the ventricles from the apex toward the aorta and pulmonary trunk.

## 2 FORMATION AND GROWTH OF THE LINEAR HEART TUBE

### 2.1 Formation of the linear heart tube

**Figure 1:**
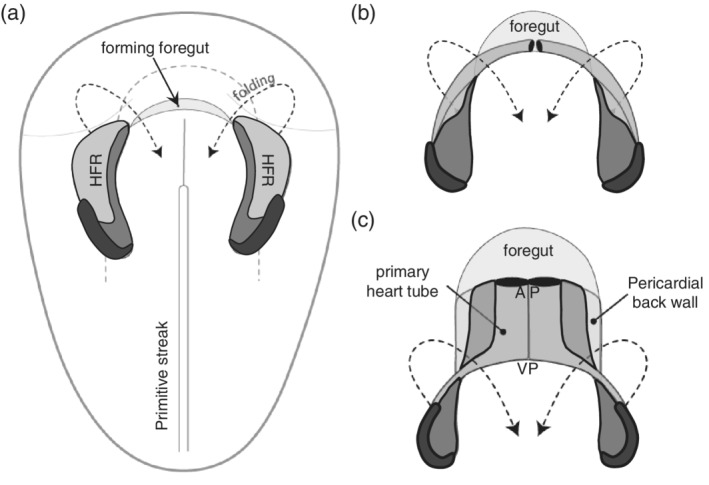
From heart forming region to primary heart tube. Panels (a)–(c) provide a schematic representation of the formation of the linear heart tube from the heart forming region (HFR), as seen from the ventral side. Within the HFR the first heart field is indicated in light gray, the second heart field in dark gray, and the third heart field in black. AP refers to the arterial pole and VP to the venous pole of the primary heart tube

Cardiac development is initiated at gastrulation at the end of the second week of human development (Carnegie Stage [CS] 7) (Figure 1). During gastrulation the two‐layered embryo‐blast becomes three‐layered, comprising the ectoderm, mesoderm and endoderm. The heart begins development from the first mesodermal cells which migrate from the site of gastrulation toward the anteriolateral border of the trilaminar embryonic disc. While migrating, these mesodermal cells will be rendered competent for differentiation toward the cardiac lineage. At the site of gastrulation, Wnt growth factors block differentiation of the mesodermal cells. While migrating anteriorly, the mesodermal cells leave the Wnt expression domain and enter a domain of active Wnt inhibition. The mesodermal cells now possess the capacity of entering the cardiac lineage. These precardiac mesodermal cells differentiate into cardiomyocytes, coordinated by Bone morphogenetic protein (BMP) growth factors are secreted by the endodermal and ectodermal cells located at the lateral border of the flat embryo at the beginning of the third week of development (CS 8). Among the first cardiac‐specific genes expressed are the transcription factors Islet1 and Nkx2.5. The area expressing these transcription factors is referred to as the heart‐forming region (also cardiac crescent) and has a horseshoe‐like shape. BMP inhibitors secreted by the neural tube regulate the medial expansion and FGF growth factors expressed by the endoderm, determine the posterior border of the of the heart forming region (Harvey, 2002; Moorman, Christoffels, Anderson, & van den Hoff, 2007; Sizarov et al., 2011; van den Hoff, Kruithof, & Moorman, 2004). At this stage differentiation proceeds quickly and the primitive cardiomyocytes start to spontaneously contract as a result of expression of (a) sarcomeric genes forming sarcomers, and (b) ion pumps and channels within the cell membrane allowing spontaneous depolarization. The contraction becomes polarized due to the electrical coupling of neighboring cells via gap junctions (Moorman et al., 2000; Moorman & Christoffels, 2003b; Tyser et al., 2016).

**Figure 2:**
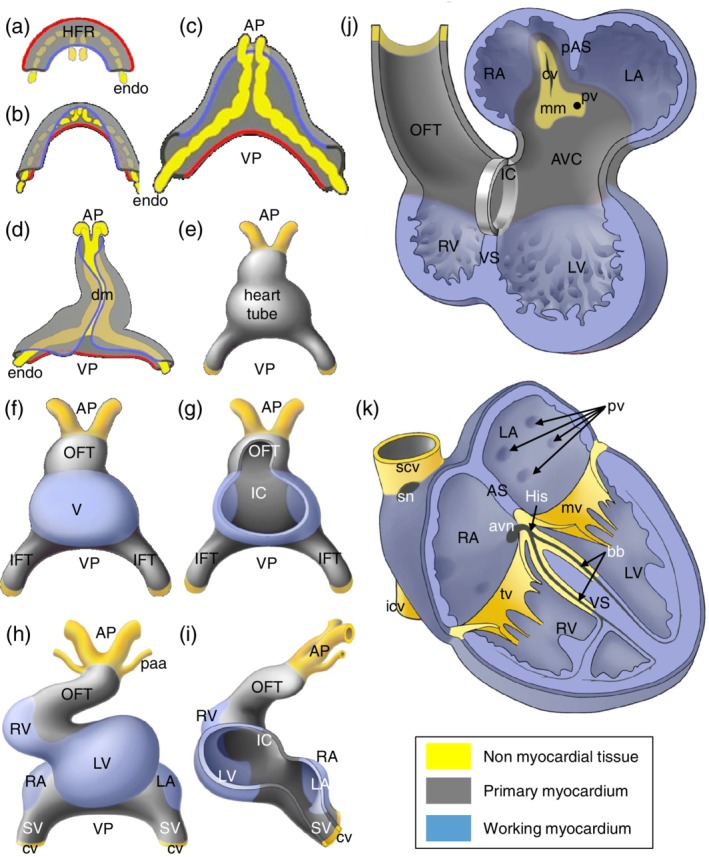
Schematic representation of the formation of the adult heart. With the onset of embryonic folding the left and right heart forming region (HFR). The yellow structures represent the endocardial cells (endo) that form the inner lining of the heart and in gray the formed primary myocardium. VP refers to the venous pole were the blood will enter the heart and AP to the arterial pole where the blood will leave the heart. For easy comparison of panels (a) through (d), a red line indicates the lateral border of the HFR and a blue line the medial border. With ongoing folding the HFR becomes positioned ventrally of the foregut (see also Figure 1). At the position where the lateral borders of the HFR meet, the linear heart tube is connected to the body wall via the dorsal mesocardium (dm) (panel d). The DM breaks due to which the heart is only attached to the body wall at the AP and VP (panel h–k). Whereas panels (a)–(d) show a dorsal view of the forming heart, panels (e)–(h) show a ventral view, illustrating the transition of linear heart tube to four‐chambered heart. Only at the ventral site of the linear heart tube the differentiation of the embryonic ventricle (V) is locally initiated. The forming working myocardium of the chambers is indicated in blue (panels f–k). Note in panel (g) that at the dorsal side of the heart tube, primary myocardium is retained, which is referred to as the inner curvature (IC). Flanking the forming ventricle, primary myocardium is retained which is referred to as the inflow tract (IFT) and outflow tract (OFT). With ongoing development, the linear heart tube loops to the right and chamber formation becomes evident (panel h). At this stage the right ventricle (RV) starts to form when primary myocardium of the OFT differentiates into chamber myocardium. Moreover, upstream of the left ventricle (LV), the primary myocardium of the IFT locally differentiates into the left atrium (LA) and right atrium (RA). In the meantime, newly differentiated cardiomyocytes are added to the lengthening heart forming the sinus venous (SV) myocardium. The heart is connected to the blood circulation at the VP via the left and right cardinal vein (cv) and at the AP via the pharyngeal arch arteries (paa). Panel (j) shows a representation of the 5 week old human heart showing the expanding (ballooning) atria and ventricles, as well as the remnants of the primary myocardium of the IFT, AVC (atrioventricular canal), IC and OFT. The forming primary atrial septum (pAS) and ventricular septum (VS) are identified. Within the LA the attachment to the body wall is identified as the mediastinal mesenchyme (mm) through which the cardinal vein (cv) and the future pulmonary vein (pv) drain into the heart. In the formed heart (panel k) the primary myocardium of the IFT and AVC has differentiated into the central conduction system, comprising the sinoatrial node (sn), the atrioventricular node (avn), the His bundle (His) and the bundle branches (bb). Within the right atrium the superior and inferior caval veins (scv and icv) drain in the RA and the pulmonary veins (pv) in the LA. Flanking the chambers, valves are formed of which only the mitral valve (mv) and the tricuspid valve (tv) are shown. The semilunar valves cannot be shown in this representation

While the precardiac mesodermal cells are formed and migrate, a subset of cells undergoes epithelial to mesenchymal transition forming endocardial cells in between the precardiac mesoderm and the endoderm (Figure 2). The endocardial cells form a network of small channels which coalesce into larger ones with ongoing development (Harris & Black, 2010). Although the myocardial and endocardial progenitors develop concomitantly, it has been demonstrated that individual cells differentiate either into endocardial cells or cardiomyocytes (Cohen‐Gould & Mikawa, 1996). Despite the fact that endocardial and endothelial cells seem to be very similar at first glance, their transcriptomes differ (Harris & Black, 2010) and mouse and zebrafish mutants exist that can form endothelial cells but no endocardial cells (Lee, Stainier, Weinstein, & Fishman, 1994).

With ongoing development, the flat embryo acquires it three‐dimensional (3D)‐shape as a consequence of folding, due to the fast growth of neural tissue at the end of the third week (CS9). Misregulation of this process can result in ectopia cordis (For review see [Gabriel et al., 2014]). During folding, the heart acquires the shape of an inverted Y with two caudolateral inlets (also venous pole) and one craniomedial outlet (also arterial pole or outflow tract). The heart tube is organized in an outer layer of two to three layers of cardiomyocytes and an inner layer of endocardial cells. The myocardial and endocardial layers are separated by an extracellular matrix (cardiac jelly). At the dorsal side the heart tube is attached to the body wall via the dorsal mesocardium. At this stage the heart tube starts to show slow peristaltic‐like contractions that are initiated at the venous pole.

## 3 THE GROWTH AND LOOPING OF THE HEART TUBE

At the beginning of the fourth week of development (CS10) the straight heart tube undergoes looping. Looping is an elusive process during which the dorsal mesocardium ruptures along its midline and the heart tube bends to the right, acquiring a C shape. With ongoing development, the bending of the heart tube becomes more complex, acquiring an S‐shape (Bayraktar & Manner, 2014; Manner, 2009).

While looping, the heart tube increases five‐fold in length due to the continuous addition of newly differentiated cardiomyocytes, rather than by proliferation of the cardiomyocytes of the heart tube (de Boer, van den Berg, de Boer, Moorman, & Ruijter, 2012a; Soufan et al., 2006). The newly differentiated cardiomyocytes are derived from a rapidly proliferating pool of mesodermal cells upstream of the venous pole of the heart. The high proliferation rate of these progenitor cells is mediated through canonical Wnt/β‐catenin signaling (Norden & Kispert, 2012; Ruiz‐Villalba, Hoppler, & van den Hoff, 2016). Fluorescent labeling of these cells in chicken demonstrated their migration into the heart tube at both the venous and arterial poles (van den Berg et al., 2009). Mouse and human cardiac development display a similar course (de Boer et al., 2012b; Sizarov et al., 2011). These cardiac progenitor cells are often referred to as second heart field cells, as opposed to first heart field cells, from which the initial heart tube is formed (Buckingham, Meilhac, & Zaffran, 2005). The distinction between these different populations is contentious, because different markers point to different borders (Moorman et al., 2007). These second heart field progenitors express the transcription factor Islet1, while being added to the heart tube. Upon differentiation into cardiomyocytes, the cells cease proliferation, coinciding with the down‐regulation of Islet1 and up‐regulation of Nkx2.5 (Buckingham et al., 2005; Cai et al., 2003). The transcription factor Tbx1 was found to be a regulator of the segregation of the second heart field cells to the inflow and outflow pole of the heart (De Bono et al., 2018; Rana et al., 2014).

## 4 FROM LINEAR TO FOUR CHAMBERED HEART

Upon rightwards looping, ventricular formation becomes evident at the outer curvature of the heart tube. At this location the cardiac jelly between the endocardial and myocardial layers disappears and myocardial protrusions, referred to as trabeculations, become evident at the luminal (endocardial) side. The cardiomyocytes of the forming ventricle start to express genes like atrial natriuretic factor (Anf/Nppa) and the gap‐junction protein Connexin 40 (Gja5) (Houweling, Somi, van den Hoff, Moorman, & Christoffels, 2002). The T‐box transcription factors Tbx5 and Tbx20 are important activators of the gene expression program in ventricular cardiomyocytes, whereas Tbx2 and Tbx3 are important repressors of the ventricular myocardium gene program in the developing atrioventricular region (Greulich, Rudat, & Kispert, 2011; Habets et al., 2002).

Prior to the appearance of the first trabeculations, mitotic spindles become oriented. Cardiomyocytes in which the spindles are parallel with the lumen of the heart tube contribute to the lengthening of the heart tube, while cardiomyocytes with mitotic spindles in the direction of the lumen will contribute to the trabeculation (Le Garrec et al., 2013; Meilhac, Esner, Kerszberg, Moss, & Buckingham, 2004). When mitotic spindle orientation is disrupted, as is observed upon deletion of atypical protein kinase C (Prkc1), trabeculation formation is affected (Passer, van de Vrugt, Atmanli, & Domian, 2016). The layout of the initial trabeculations is defined by the concerted action of endocardially expressed Notch1 and Neuregulin 1. The endocardium becomes sculptured and forms domes filled with cardiac jelly. At the side where the endocardium contacts the myocardium, Nrg1 expressed by endocardial cells can interact with ErbB2/ErbB4 expressed by the cardiomyocytes (Del Monte‐Nieto et al., 2018). Notch1 regulates the proliferation of the cardiomyocytes that are added to the base of the forming trabeculations in the domes (Del Monte‐Nieto et al., 2018; Grego‐Bessa and others, 2007). Bmp10 expression in the trabecular cardiomyocytes is an important regulator of trabecular growth, shown by the facts that (a) deletion of Bmp10 results in hypotrabeculation (Chen et al., 2004) and (b) overexpression of Bmp10 in hypertrabeculation (Pashmforoush et al., 2004). The expression of the cell cycle inhibitor (Cdkn1c) in the cardiomyocytes of the trabeculations suggests active inhibition of proliferation of these cells (Kochilas, Li, Jin, Buck, & Epstein, 1999). Within the next six days of mouse development the ventricle increases 100‐fold in volume, due to active cardiomyocyte division of the ventricular wall, resulting in outer ventricular wall thickening and increase in trabecular length. The lengthening of the trabeculations is the result of addition of cardiomyocytes to their base, rather than proliferation at their tips (de Boer, van den Berg, de Boer, et al., 2012a).

Although the initially formed trabeculations are long and slender compared to the compact outer myocardial layer, they become relatively short and thick with ongoing development. It should be noted that the absolute length of these adult thick trabeculations is much longer than the embryonic trabeculations. This change in appearance of the trabeculations is referred to as compaction. This process is complete by embryonic day (E)14.5 in mice and at about eight weeks of development (CS22) in human (Sedmera, Pexieder, Vuillemin, Thompson, & Anderson, 2000). How these local differences in proliferation are molecularly regulated is largely unknown. A number of cell cycle regulators were shown to result in hypoplastic ventricle upon functional impairment in mice (Berthet et al., 2006; Koera et al., 1997; Kozar et al., 2004; Moens, Stanton, Parada, & Rossant, 1993). Interestingly, in mice in which N‐myc is deleted, the compact layer remains thin (Charron et al., 1992; Moens et al., 1993). In human, mutations in these genes might underlie nonventricular compaction.

During ventriclular development and heart tube lengthening, the atria begin to differentiate at the inflow region (Yutzey, Rhee, & Bader, 1994). The atria are formed symmetrically, but have a left–right identity from the outset. The left‐sided identity is imposed by the transcription factor Pitx2c. As a consequence, the absence of Pitx2c expression results solely in right atrial identity and thus two morphologically identical atria, also referred to as right atrial isomerism. Vice versa, ectopic expression of Pitx2c at the right side, results in two morphologically left atria, that is, left atrial isomerism (Mommersteeg and others, 2007b). Like the forming ventricular myocardium the developing atrial myocardium is also marked by ANF and Cx40 expression. The initially formed atria are retained in the heart as the trabeculated, atrial appendages (Christoffels et al., 2000).

The smooth‐walled myocardium found in the atria is added later during development. The confluence of the bilaterally developing superior and inferior cardinal veins drain into the respective left and right common cardinal veins which in turn drain into the early heart. These regions are referred to as the left and right sinus horns. Both common cardinal veins are accreted from the flanking body wall adjacent to the venous pole. While the pericardial cavity expands the common cardinal veins are absorbed into the pericardial cavity, their walls become muscularized. This myocardial part upstream of the atria is referred to as the sinus venosus. The myocardial progenitor cells forming the sinus venosus are different from the first and second heart field cells and often referred to as the third heart field. These progenitors are Tbx18‐positive, Nkx2.5 and Wt1 negative, and their proliferation is regulated by canonical Wnt signaling (Norden et al., 2010; Norden, Grieskamp, Christoffels, Moorman, & Kispert, 2012). This newly formed myocardium along the right common cardinal vein will become the dorsal aspect of the right atrium, in between the entrance of the superior and inferior caval vein, and is referred to as the sinus venarum in the adult heart. The left common cardinal vein becomes the coronary sinus, via which the coronary venous circulation drains into the right atrium. The connection to the left superior and inferior cardinal veins regresses and becomes the Ligament of Marshall. The left superior cardinal vein, contributing in the adult to the left internal jugular vein, drains via the brachiocephalic vein into the superior caval vein, which originates from the right superior cardinal vein. The orifices of the veins in the right artrium are guarded by valves; the valve that flanks the orifice of the superior caval vein is the venous valve, the inferior caval vein the Eustachian valve and the coronary sinus the Thebesian valve. How the formation and remodeling of these structures is molecularly regulated is largely unknown (Norden & Kispert, 2012; van den Hoff et al., 2004).

In the dorsal wall of the adult left atrium a large portion of smooth‐walled myocardium can be found, referred to as pulmonary myocardium. This myocardium is derived from second heart field cells located in the dorsal mesocardium at the venous pole of the heart. Within this mesenchyme and the mesenchyme, which surrounds the embryonic foregut contiguous with the dorsal mesocardium, a vascular plexus is formed which contributes to the lung vasculature. At about five weeks of development (CS13), a single vessel forms through the dorsal mesocardium which connects this vascular plexus around the foregut with the atrium. Semaphorin 3d (Sema3d) is expressed in the mesothelial cells covering the dorsal mesocardium and as such flanking the tissue through which the pulmonary vessels are formed. Sema3d is thought to act as a repulsive guidance molecule to constrain and direct the developing pulmonary venous endothelial cells toward the atrium. Sema3d‐mediated endothelial repulsion is most probably mediated through Neuropilin 1 (Nrp1) expressed on the endothelial cells. In Sema3d‐null mice the pulmonary venous endothelial cells display abnormal invasion of the dorsal mesocardium resulting in an abnormality referred to as abnormal pulmonary venous connection or return (APVC) (Aghajanian et al., 2014; Degenhardt et al., 2013]. APVC refers to a spectrum of abnormalities in which the pulmonary vein is not connected to the left atrium, but to the right atrium directly, or indirectly through the coronary sinus or the superior or inferior caval veins. This was underscored in sequencing SEMA3D in patients with APVC, identifying a mutation in SEMA3D that affects the function of SEMA3D (Degenhardt et al., 2013). Furthermore, a Scottish family with APVC was identified with a genomic alteration located in 4p12 (Bleyl et al., 2006), pointing perhaps to regulation of this developmental process by other genes. Although guided growth of the pulmonary vein toward the atrium seems to play an important role, it should be noted that, anatomically, the initial pulmonary venous return is a midline structure. Since the primary atrial septum develops at the right side of the pulmonary orifice, the pulmonary vein becomes incorporated into the left atrium.

Subsequent to the connection of the pulmonary vein to the atrium, a myocardial mantle forms around the pulmonary vein and its bifurcations. Mesenchymal cells flanking the pulmonary venous endothelium in the dorsal mesocardium differentiate into cardiomyocytes. The population is, in contrast to the myocardium formed around the caval veins, derived from a Tbx18‐negative, and Nkx2.5‐ and Isl1‐positive progenitor population. These newly formed cardiomyocytes initiate rapid proliferation and migrate along the pulmonary veins forming a myocardial sleeve. Interestingly, in the absence of functional Pitx2c results in under population of the walls of the pulmonary veins (Mommersteeg et al., 2007a). In mice, the myocardial sleeve is found to extend deep into the lungs, up to the fifth bifurcation. In human, however, this sleeve only develops to the extent of the second bifurcation, and while this sleeve is being formed, it is also being taken up into the dorsal wall of the left atrium. As a consequence, four pulmonary orifices are found in the left atrium and an extensive amount of smooth‐walled myocardium in between these orifices.

## 5 DEVELOPMENT OF THE CONDUCTION SYSTEM

The timing and coordination of muscular contraction in the adult heart is coordinated by the sinus node, AV node, AV bundle, bundle branches and the peripheral conduction system (Purkinje fibers). The cardiac conduction system develops hand in hand with the forming cardiac chambers.

At the end of the second week of development the mesodermal cells differentiating into cardiomyocytes cells begin to become electrically competent and begin to twitch. In the beginning of the third week of development (CS9‐10), when the linear heart tube has just formed, peristaltic contraction waves travel from inflow to outflow regions. An ECG can already be recorded at this stage and has a sinusoidal morphology (Christoffels, Smits, Kispert, & Moorman, 2010; Hoff, Kramer, DuBois, & Patten, 1939). It should be noted that at this stage of development, there is no morphologically distinguishable cardiac conduction system. The slow and long lasting contraction is the result of poorly electrically coupled cardiomyocytes, and at the same time the sarcomers and sarcoplasmic reticulum have yet to fully develop. The polarity of this contraction wave is due to the expression of the hyperpolarization activated pacemaker channel Hcn4 in the cardiomyocytes located at the inflow region of the heart tube (Mommersteeg and others, 2007b). Hcn4 is responsible for the spontaneous depolarizing “funny” current, a major component of pacemaker activity. Since pacemaker activity is always found at the most distal myocardial border of the inflow, the newly differentiated cardiomyocytes at the distal border of the inflow possess dominant pacemaker activity, determining the contraction rate of the heart (Moorman, Christoffels, & Bakker, 2011).

When chamber formation starts around three weeks of human development (CS10), the myocardium of the forming atrial and ventricular chambers conducts the depolarizing wave faster than the remnants of primary myocardium (F. and others, 1992), which flank the chambers; the venous pole, atrioventricular canal and outflow tract. Besides the relative fast‐conduction in these atrial and ventricular cardiomyocytes, their differentiating sarcomeres and sarcoendoplasmatic reticulum allow faster and more efficient contractions. The difference in contraction characteristic of the chamber and primary cardiomyocytes prevents backflow of blood and enabling the propulsion of blood without valves. Cardiomyocytes of the sinus venosus have the highest frequency of automaticity, have poorly developed sarcomeres and are poorly electrically coupled to one another and to the flanking atrial cells. These characteristics allow them to build up an electrical charge and drive the depolarization of the cardiomyocytes in the downstream compartments (Moorman & Christoffels, 2003a). At this developmental stage an ECG can be registered that resembles the ECG of the adult (Hoff et al., 1939). Despite the absence of a central conduction system, this adult‐like ECG can be recorded due to the alternating arrangement of cardiac compartments with different intrinsic properties.

## 6 THE SINUS NODE

An excellent in‐depth review discussing the transcriptional networks underlying the development of the cardiac conduction system has recently been published (van Eif, Devalla, Boink, & Christoffels, 2018). The sinus node develops within the myocardium added to the venous pole and is characterized by the expression of Tbx18 and the absence of Nkx2.5 expression (Mommersteeg et al., 2010; Wiese et al., 2009). The remainder of the sinus venous myocardium differentiates into atrial myocardium and starts to express Nkx2.5 and the chamber myocardial markers, like Cx43 (Gja1) and ANF (Nppa) (Mommersteeg and others, 2007b). The expression of Nkx2.5 is under the control of the transcription factor Shox2 (Blaschke et al., 2007; Espinoza‐Lewis et al., 2009). Tbx3 is expressed in the developing sinus node, which suppresses the chamber‐specific gene expression program. Tbx3 retains its expression in the adult sinus node and when ectopically overexpressed in atrial working myocardium, Hcn4 expression is activated and ectopic pacemaker function can be observed (Hoogaars et al., 2007). The formation of the sinus node at the right side is regulated by the transcription factor Pitx2. Pitx2 is only expressed in the left sinus horn myocardium, in which it suppresses right‐sided sinus node differentiation (Mommersteeg et al., 2010; Wiese et al., 2009).

## 7 ATRIOVENTRICULAR NODE AND BUNDLE BRANCHES

The AV node and bundle branches develop within the myocardium of the AVC, the cardiac compartment in between the forming atria and ventricles. The atrioventricular canal (AVC) myocardium expresses Bone Morphogenetic Protein 2 (Bmp2) which is key in maintenance of the expression of Tbx2 and Tbx3 in the AVC myocardium. These two transcription factors actively repress the chamber specific gene expression program in the AVC myocardium (Aanhaanen et al., 2011; Christoffels et al., 2010; Singh et al., 2012; Stefanovic et al., 2014). The importance of Bmp‐signaling is crucial in this process as AVC‐specific deletion of the Bone morphogenetic protein receptor 1A (Bmpr1a) leads to impaired atrioventricular node and annulus fibrosis development (Gaussin et al., 2005; Stroud et al., 2007). AVC development is further confined by Notch‐signaling (Rentschler et al., 2011) and transcriptional regulation through Tbx20 (Singh et al., 2009).

A cluster of cells on top of the forming ventricular septum will not follow the chamber myocardial gene expression program, but retain their primary myocardial phenotype. This group of cells will differentiate into the atrioventricular (His) bundle. Although this was already concluded by Keith and Flack in 1906 (Keith, Aberb, Eng, Flack, & Oxon, 1906), it took till the end of the century to visualize and follow these cells immunohistochemically using the GlN2 antibody (Lamers et al., 1992; Wessels et al., 1992) and later by expression studies of Tbx3 (Bakker et al., 2008; Hoogaars et al., 2004). The cardiomyocytes of the atrioventricular bundle and bundle branches express Connexin 40 (Gja5) in contrast to the cardiomyocytes of the atrioventricular node. Expression of Gja5 warrants the fast conduction of the electrical impulse through the bundles (Davis, Rodefeld, Green, Beyer, & Saffitz, 1995). Their development is regulated through a molecular network including Nkx2.5, Tbx5 and Id2. Interestingly, heterozygous deletion of Nkx2.5 results in mice in an underdeveloped atrioventricular bundle and atrioventricular block (Moskowitz et al., 2004; Moskowitz et al., 2007).

## 8 PERIPHERAL VENTRICULAR CONDUCTION SYSTEM

The electrical impulse traveling through the bundle branches is propagated to the individual cardiomyocytes via the Purkinje fibers. The ventricular conduction system is fast‐conducting by virtue of the gap junctions Gja1 and Gja5. Permanent genetic labeling of Gja5‐positive cells during heart development has shown that before E14.5 in mouse all ventricular cardiomyocytes are positive and become subsequently restricted to the cardiomyocytes located subendocardially in the tips of the ventricular trabecules, forming the peripheral ventricular conduction system (Miquerol et al., 2010). The electrical impulse is propagated from the endocardially located cardiomyocytes to the epicardially located cardiomyocytes via their intercellular connections. Although differential growth of the compact and trabecular layer is suggested to underlie the confinement of the peripheral ventricular conduction system (de Boer, van den Berg, Soufan, et al., 2012b), the molecular regulation of this process has yet to be established.

## 9 SEPTATION

**Figure 3:**
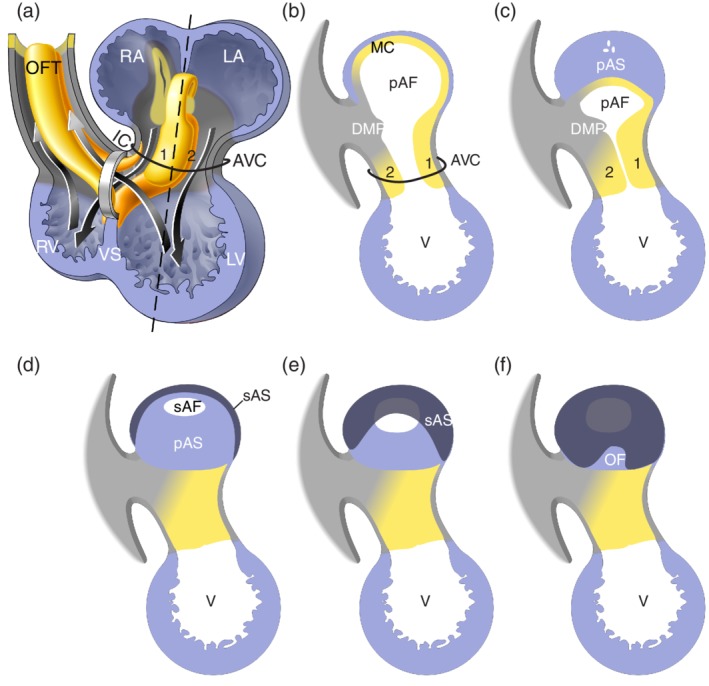
Schematic representation of septation. Panel (a) shows the same schematic drawing of the four chamber‐forming heart as Figure 2(j), in which now the major cushions have been added within the atrioventricular canal (AVC) and outflow tract (OFT) and the left and right bloodstreams through the heart is indicated. The ventricular foramen is located between the distal edge of the ventricular septum (VS) and the inner curvature (IC). Noteworthy is that the left bloodstream passes the ventricular foramen during systole, while the right bloodstream already passes through the ventricular foramen during diastole. Panels (b)–(f) illustrate atrial septation and show the cutting edge (sagittal sections) at the level of the dashed line in panel (a). For easy comparison the cushions are numbered. The mesenchymal complex formed by the anterior (1) and posterior (2) atrioventricular cushion, the extension of the anterior cushion over the roof of the atrium (MC), and the extracardiac mesenchyme that protrudes into atrial lumen (DMP), surround the connection between the left (LA) and right atrium (RA). With expansion of this complex the primary atrial foramen (pAF) becomes smaller and eventually closes forming the primary atrial septum (pAS). Prior to closure of the pAF, the secondary atrial foramen (sAS) is formed. Within the RA the secondary atrial septum (sAS) folds down from the atrial wall into the lumen and covers the pAS partly and the pAF completely. The uncovered part of the pAS is recognized as the oval fossa (OF) (Modified from Sylva et al., 2014)

Septation of the heart is a complex process during which the initial single bloodstream is physically separated into a systemic and pulmonary stream (Figure 3). Half of the cardiac congenital abnormalities are accounted for by septational abnormalities, which range from nonpathological to embryonic, fetal or neonatally lethal.

Septation starts with the expansion of the extracellular matrix between the endocardial and myocardial cell layers in the atrioventricular canal and the outflow tract. This extracellular matrix, secreted majorly by surrounding cardiomyocytes, is also referred to as cardiac jelly. The extracellular matrix deposits become molded into four major cushions; the posterior and anterior atrioventricular cushions in the AVC and the parietal and septal outflow cushions, often referred to as ridges, of the outflow tract (OFT). The cushions initially become populated by endocardium‐derived mesenchymal cells (de Lange et al., 2004; Markwald, Fitzharris, & Manasek, 1977). The endocardial‐to‐mesenchymal transition of the endocardium overlaying the cushions is regulated by Notch, Bmp and Tgfb‐signaling (For review see [Garside, Chang, Karsan, & Hoodless, 2013; MacGrogan, Luna‐Zurita, & de la Pompa, 2011; Mercado‐Pimentel & Runyan, 2007; van Wijk, Moorman, & van den Hoff, 2007]). The OFT cushions, in addition to the endocardially derived cells, also become populated by neural crest derived cells (de Lange et al., 2004; Lincoln, Alfieri, & Yutzey, 2006). The cushions expand into the lumen, meet and fuse, separating the left and right bloodstreams. It should be noted that the cushions also contribute to the valves in the atrioventricular canal and the outflow tract, which will be described below.

## 10 ATRIAL SEPTATION

Atrial septation starts with the formation of the primary atrial septum during the fourth week of developmental (CS12). The primary atrial septum forms as a result of proliferation of atrial cells. The growing atrial septum has the shape of a crescent and its leading edge is covered with a mesenchymal cap. Anteriorly this cap is contiguous with the anterior atrioventricular cushion and at the posterior site with the dorsal mesocardial protrusion (DMP) and the posterior atrioventricular cushion. In humans the DMP is anatomically more pronounced than in mice (Snarr, Kern, & Wessels, 2008; Snarr, Wirrig, Phelps, Trusk, & Wessels, 2007b; Wessels et al., 2000). Genetic lineage tracing has shown that the mesenchymal cells found in the DMP are derived from the second heart field (Goddeeris et al., 2008; Snarr et al., 2007a). The formation of the DMP is molecularly regulated by Sonic Hedgehog (Shh), Wnt and Bmp (Briggs et al., 2013; Briggs et al., 2016; Goddeeris et al., 2008). The mesenchymal complex comprised of the two major atrioventricular cushions, the mesenchymal cap of the primary atrial septum and the DMP, encircles the primary atrial foramen. During the sixth week of development (CS16) this primary atrial foramen closes by the fusion of the mesenchymal components of this complex. If this complex does not fuse completely or partly, a range of defects is observed from an atrial septal defect type one (ASD‐I) to a partial or complete atrioventricular septal defect (AVSD). Interestingly, in mouse models for Down syndrome the DMP is absent and in 30–50% of children with Down syndrome are diagnosed with an AVSD (Blom, Ottenkamp, Wenink, & Gittenberger‐de Groot, 2003; Briggs, Kakarla, & Wessels, 2012; Webb, Anderson, Lamers, & Brown, 1999).

During development the pulmonary circulation needs to be largely by‐passed during intra‐uterine development. To this end, a new foramen, the secondary atrial foramen, is formed in the primary atrial septum prior to the closure of the primary atrial foramen. From the end of the sixth week of development (CS17) onwards, the muscular wall of the right atrium folds down into the lumen of the right atrium to form the secondary atrial septum. The secondary atrial septum folds deep into the atrium and covers the entire secondary foramen. At birth when the lungs become functional and the ductus arteriosus closes, the blood pressure in the right atrium lowers, whereas the pressure at the left side hardly changes. Due to this difference in blood pressure between the right and left atrium, the primary atrial septum is pushed against the secondary atrial septum, preventing blood flow from the right to the left side. In two thirds of the human population the two septa subsequently fuse, whereas in the other one third the communication remains a situation referred to as a patent foramen ovale. When the secondary atrial septum does not form or is hypoplastic, the result is a secondary atrial septum defect or atrial septal defect type two (ASD‐II).

## 11 VENTRICULAR SEPTATION

The septum dividing the left and right ventricle in the formed heart is composed of the myocardial ventricular septum and the membranous septum.

When the right and left ventricles form the cells in between will form the myocardial ventricular septum. The ventricular septum lengthens by a process called apposition, meaning that cells are added to the septum at its base (Harh & Paul, 1975). In human this process starts in the fourth week of development (CS12) (Sizarov et al., 2011). It is thought that when apposition is aberrant that a hole or multiple holes will be formed in the ventricular septum. These left–right connections are referred to as muscular ventricular septal defects (VSDs).

The lumen in between the top of the interventricular septum and the inner curvature is referred to as the ventricular foramen. Because the bloodstream is laminar, particles introduced into the blood follow a specific path without lateral mixing (Hogers, deRuiter, Baasten, Gittenberger‐de Groot, & Poelmann, 1995). These analyses showed that the blood flow is already separated into a left‐ and right‐sided circulation prior to septation, and that blood from the right atrium passes through the ventricular foramen into the right ventricle during diastole and blood from the left ventricle passes through the ventricular foramen into the future aortic stream during systole. From this analysis it is obvious that the ventricular foramen never closes but becomes septated (de Jong, Moorman, & Virágh, 1997). The ventricular foramen is divided in a left and right component by the two major AV cushions and the septal OFT ridge, becoming the membranous septum in the adult human from the second half of the sixth week of development (CS19) onward. One can now also understand why this septum has an interventricular and an atrioventricular portion, and why perimembranous or inlet VSDs are present as a spectrum (Lopez et al., 2018).

## 12 SEPTATION OF THE OUTFLOW TRACT

The outflow tract is a myocardial tube that runs from the developing ventricles to the aortic sac, which is embedded in the pharyngeal arches and is connected to three paired symmetrical pharyngeal arch arteries. The pharyngeal arch arteries remodel in to the arterial pole of the heart which already resembles the postnatal configuration at the end of the eight week of development (CS23) (Rana, Sizarov, Christoffels, & Moorman, 2013).

Initially, the myocardial OFT increases in length by the addition of newly differentiated cardiomyocytes to its distal border (Rana et al., 2007; Webb, Qayyum, Anderson, Lamers, & Richardson, 2003). These cardiomyocytes are derived from the second heart field (Kelly & Buckingham, 2002; Sizarov et al., 2012). From the fifth week of development (CS14) onward the myocardial OFT becomes shorter, due to the incorporation of the proximal OFT myocardium as part of the right ventricle. In the adult the distal myocardial border is found halfway at the level of the semilunar valves and below the coronary orifices. As a consequence, the OFT has a nonmyocardial portion in between its distal of myocardial border and the border of the pericardial cavity. This nonmyocardial portion of the OFT will become the intrapericardial part of the aorta and pulmonary trunk (Rana et al., 2007; Sizarov et al., 2012).The cells of the nonmyocardial portion originate from both the second‐heart field and the cardiac neural crest (Cai et al., 2003; Jiang, Rowitch, Soriano, McMahon, & Sucov, 2000; Zhou et al., 2017).

Septation of the OFT starts at its distal border in the sixth week of development (CS16) and proceeds in a proximal direction, to its completion a week later (CS18) (Sizarov et al., 2012). The OFT cushions lay in a spiraling fashion, reflecting the course of the aortic and pulmonary streams in the adult (Sizarov et al., 2012; Ya et al., 1998). At the start of fusion of the OFT cushions, the proximal portion of the cushions is invaded by endocardial derived cells and by cardiac neural crest cells at its distal border. The migration of the neural crest cells into the heart is a complex process that is regulated by Wnt, Bmp, Fgf, and Semaphorin signaling (For review see [Stoller & Epstein, 2005]. Invasion of neural crest cells becomes evident with the formation of a protrusion of pharyngeal mesenchyme, termed the aorticopulmonary septum, that grows into the aortic sac and connects distally to the fused OFT cushions. The aorticopulmonary septum ensures that the aortic blood stream is guided into the left fourth pharyngeal arch artery and the pulmonary blood stream into the left and right sixth pharyngeal arch arteries (Anderson et al., 2009; Anderson et al., 2012; Rana et al., 2013). In the distal portion of the OFT, the cardiac neural crest cells form condensed pillars in the cushions and in the proximal portion of the OFT cushions, where they become dispersed (Waldo, Miyagawa‐Tomita, Kumiski, & Kirby, 1998). In the distal portion of the OFT the neural crest cells will predominantly form the facing part of the walls of the aorta and pulmonary trunk. In the proximal portion of the OFT the cushions form upon fusion of the outlet septum, in which a large part of the mesenchyme disappears by apoptosis and is replaced by cardiomyocytes. A process referred to as myocardialization (van den Hoff et al., 1999; Ya et al., 1998). The myocardial outlet septum changes during the commitment of the aorta to the left ventricle largely into the freestanding subpulmonary infundibulum. In the adult right ventricle, remnants of the OFT myocardium and myocardial outlet septum are recognized as smooth‐walled myocardium distal of the trabecular component (Anderson et al., 2009; Anderson et al., 2012). Even in the adult, this smooth‐walled myocardium still has characteristics of the primary myocardium due to which it can serve as a source for arrhythmias in cardiac disease (Boukens et al., 2013).

When the OFT cushions do not fuse over their entire length, an anomaly known as a persistent truncus arteriosus or common arterial trunk arises. When the fusion defect is limited to the proximal OFT cushions, this results in a subarterial or outlet VSD (Anderson et al., 2012; Okamoto, Akimoto, Hidaka, Shoji, & Sumida, 2010). A transposition of the great arteries is found when the cushions are laid down in a parallel fashion rather than in a spiraling course. In a transposition of the great arteries the aorta and pulmonary trunk are situated next to each other in a frontal plane and at birth two separate blood circulatory systems are formed which is not compatible with life (Costell et al., 2002; Kirby, 2002). Because cardiac neural crest provide cells to the OFT and these cells secrete growth factors essential for normal development of the surrounding tissue, no or a limited number of cells invade the OFT a spectrum of abnormalities ranging from a common trunk, to pulmonary atresia and double outlet right ventricle arises (Hutson & Kirby, 2003; Stoller & Epstein, 2005). Moreover, when fusion of the aorticopulmonary septum and the proximal OFT cushions is impaired, this is recognized as an aorticopulmonary window (Anderson et al., 2012; Sizarov et al., 2012; Rana et al., 2013.

## 13 THE CARDIAC CONNECTIVE TISSUES

Cardiomyocytes make up 90% of the volume of the adult heart muscle, but only comprise 50% in total cell number (Banerjee, 2007; Pinto et al., 2016). Of the nonmyocytes, more than half of the cells are endocardial/endothelial cells, approximately one third are fibroblasts and less than 10% are hematopoietic‐derived cells (Pinto et al., 2016). Already one month after birth the maximum number of cardiomyocytes in the human heart has been reached, being approximately 3.2 × 10^9^ ± 0.75 × 10^9^ cardiomyocytes (Bergmann et al., 2015). From first month after birth onward, the increase in size of the heart is almost exclusively due to a volume increase of the cardiomyocytes (hypertrophy), being almost 10‐fold up to an age of 20 years (Bergmann et al., 2015). Moreover, only approximately 50% of the cardiomyocytes undergo a single round of cell division during an adult lifespan (Bergmann et al., 2009; Bergmann et al., 2015) and hardly any cardiomyocytes are supplement from a stem cell population (Jesty et al., 2012; van Berlo et al., 2014). A recent review extensively discusses this phase of heart development (Gunthel, Barnett, & Christoffels, 2018).

Cardiac fibroblasts have long been neglected functionally, but in the last decades their importance under normal physiological and pathological condition has started to become evident. Analysis of the cardiac fibroblast population in the heart also showed that they are not a single homogeneous group. Since the introduction of genetic lineage marking systems, it has become evident that fibroblasts are not only derived from the epicardial cells but also from endocardial, circulating and hematopoietic cells. In depth reviews on this topic have been recently published (Ivey & Tallquist, 2016; Swonger, Liu, Ivey, & Tallquist, 2016; Tallquist & Molkentin, 2017).

## 14 EPICARDIUM AND ITS DERIVATIVES

The epicardium is derived from the proepicardium, a villous structure found immediately upstream of the heart tube at the beginning of the fourth developmental week (CS10). Within days (CS11) the villi contact the dorsal outer surface of the AVC and from that point cells spread and start to cover the entire heart with epicardium, a process complete at the end of the sixth week of development (CS16) (Hirakow, 1992). A subset of epicardial cells undergoes epithelial to mesenchymal transition giving rise to a mesenchymal layer between the epicardium and myocardium. Although not fully analyzed it is general contention that the epithelial to mesenchymal transition observed in the epicardium is largely comparable to epithelial to mesenchymal transition in the cushions. This formed sub‐epicardial mesenchyme either remains or contributes to (1) the coronaries, (2) cardiac fibroblasts, (3) annulus fibrosus and (4) valve leaflets (Perez‐Pomares & de la Pompa, 2011; Wessels et al., 2012).

Initially, it was thought that the entire coronary vessel tree would be formed from epicardial‐derived cells by vasculogenesis and subsequently angiogenesis. However, studies using genetic lineage tracing experiments revealed that this appeared to be more complex. Epicardial‐derived cells contribute the coronary smooth muscle cells and adventitial fibroblasts. The coronary endothelium was found to be formed from endocardial‐derived cells, endothelial cells of the sinus venosus, and a limited contribution of epicardial‐derived cells. In‐depth reviews on this topic were recently published (Perez‐Pomares & de la Pompa, 2011; Sharma, Chang, & Red‐Horse, 2017). When development of the coronary tree reaches completion, the two coronary arteries form by angiogenesis which tap into the lumen of the aorta (Bogers, Gittenberger‐de Groot, Poelmann, Péault, & Huysmans, 1989). The signals that promote the coronary arteries to grow toward the aorta and perforate the aortic wall are unknown, as yet. Erroneous connections are found when development of the outflow tract is affected (Theveniau‐Ruissy et al., 2016). It is, however, not known whether these primary or secondary defects are due to abnormal OFT development. Congenital coronary artery anomalies are frequently observed and of major importance in cardiology and cardiac surgery, because of their association with myocardial ischemia and sudden death. An extensive description of these abnormalities has recently been published (Perez‐Pomares et al., 2016).

Another part of the epicardial‐derived mesenchyme migrates into the myocardium forming fibroblasts. Genetic labeling studies in mouse have shown that the epicardial derived fibroblasts appear and populate the developing compact layer of the ventricular myocardium. It is only at the end of gestation that the epicardial derived fibroblasts also populate the trabeculations (Wessels et al., 2012). Fibroblasts regulate myocardial proliferation through growth factors, cytokines, and extracellular matrix proteins. It has been found that myocardially expressed beta1‐integrin mediates this proliferative affect via Erk1/2 and PI3K/Akt intracellular signaling pathways (Ieda et al., 2009).

Genetic labeling of the epicardial cells also revealed that the annulus fibrosis is derived from this cell population and a part of the cells of the atrioventricular valve leaflets (Aanhaanen et al., 2010; Wessels et al., 2012; Zhou and others, 2010). Their contribution to the valves will be discussed below.

## 15 DEVELOPMENT OF THE VALVES

**Figure 4:**
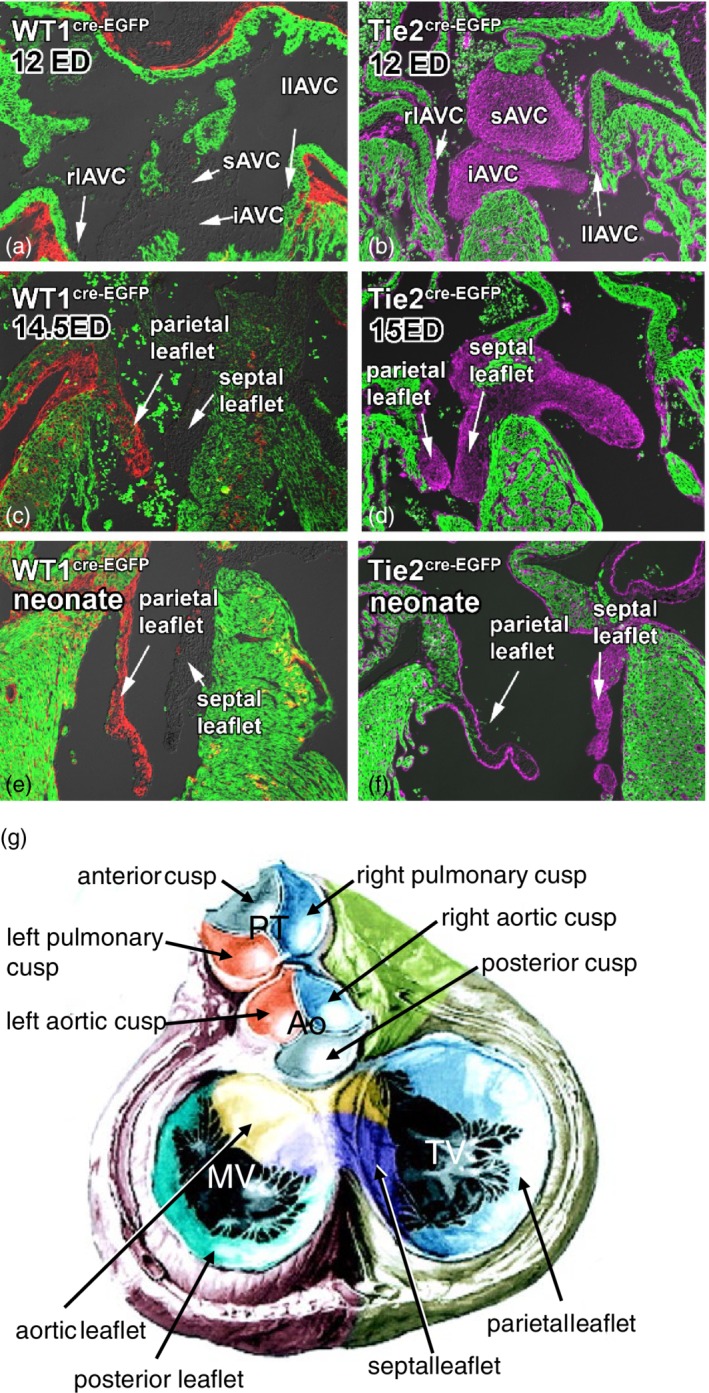
Formation of the valves. Panels (a)–(f) show the formation of the atrioventricular valves. Genetic lineage tracing shows the contribution of the endocardially‐derived (panels b, d, and f) and epicardially‐derived (panels a, c, and e) cells to the cushions and subsequent valve leaflets. The myocardium of the heart is shown in green and the epicardially‐derived cells labeled using the WT1(BAC)‐Cre in red and the endocardially‐derived cells using Tie2‐Cre in magenta (Modified from Wessels et al., 2012). Panel (g) shows a schematic summary of the origin of the cellular contributions to the mitral, tricuspid and semilunar valves. In the pulmonary trunk The left cusp in both the pulmonary trunk and aorta is derived from the septal OFT cushion (blue). The right cusp in both the pulmonary trunk (PT) and aorta (Ao) is derived from the parietal OFT cushion (orange). The anterior cusp in the pulmonary trunk is derived from the right intercalated ridge and in the aorta the posterior cusp is derived from the left intercalated ridge (gray). The aortic or anterior leaflet of the mitral valve (MV) and the septal leaflet of the tricuspid valve (TV) are derived from the inferior (blue) and posterior (yellow) atrioventricular cushions (iAVC and sAVC, respectively). The posterior leaflet of the MV is derived from the left lateral atrioventricular cushion (llAVC, green) and the septal leaflet of the TV from the right lateral atrioventricular cushion (rlAVC, light blue). From the data shown in panels (a)–(f), one can infer that the posterior MV leaflet and the parietal leaflet of the TV comprise predominantly of epicardially‐derived cells and both the aortic leaflet of the MV and the septal leaflet of the TV of endocardially‐derived cells. (Modified from Lamers & Moorman, 2002)

The valves are formed from a part of the four major cushions, as described in the section on septation (Figure 4). In both the AVC and OFT minor cushions are formed, the so‐called lateral AV cushions and the intercalated OFT ridges (de Lange et al., 2004; de Vlaming et al., 2012; Lamers & Moorman, 2002; Snarr et al., 2008; Wessels et al., 2012). Although still to be experimentally evaluated, the development of the minor cushions and intercalated ridges is similar to that of the major cushions. As summarized in Figure 4, genetic marking of the epicardial or endocardial cell contribution to the AV cushions has revealed that the leaflet of the mitral valve that is attached to the left ventricular free wall is derived from the left lateral AV cushion; the leaflet that is attached to the ventricular septum is derived from the major cushions; the leaflet of the tricuspid valve that is attached to the right ventricular free wall is derived from the right lateral AV cushion; the leaflet that is attached to the ventricular septum is derived from the major cushions (de Lange et al., 2004; Lockhart, Phelps, van den Hoff, & Wessels, 2014; Wessels et al., 2012). Interestingly, epicardially‐derived cells populate both the left and right lateral AV cushions, displacing the endocardially‐derived cells (Wessels et al., 2012). Although, the semilunar valves in the aorta and pulmonary trunk are virtually devoid of epicardial‐derived cells (unpublished personal observations), they do receive an extracardiac contribution from the cardiac neural crest (de Lange et al., 2004; Jiang et al., 2000). The cardiac neural crest‐derived cells are destined to become the part of the semilunar valve leaflets that is attached to the aortic or pulmonary trunk wall. The semilunar valve leaflets that are attached to the facing walls of the aorta and pulmonary trunk are derived from the major OFT cushions, whereas the semilunar leaflets that are attached to the nonfacing part of the walls of the aorta and pulmonary trunk are derived from the left and right intercalated ridges, respectively. The function of these extracardiac cell populations in the valve leaflets is, as yet, not known.

To become free hanging valve leaflets the cushions need to be detached from the underlying supporting tissue. The part of the cushions forming the aortic leaflet of the mitral valve protrudes from its outset, into the left ventricular lumen, never have being in contact with myocardium. This differs to the septal leaflet, the last to form in the eight week of development (CS23), of the tricuspid valve which has to detach by apoptosis from the underlying myocardium. The anterior and posterior leaflets of the tricuspid valve and the parietal leaflet of the mitral valve do not detach from the underlying myocardium by apoptosis, but by excavation or ventricularization; the ventricular lumen expands behind the cushions creating a space and liberating the valve leaflets. This process starts in developmental week seven (CS19). Cardiomyocytes left behind during leaflet formation, are removed by apoptosis during subsequent development (de Lange et al., 2004). While the valve leaflets are forming the distal part of the embryonic valve differentiates into the chordae tendineae that prevent the leaflets from prolapsing into the atria, during contraction of the ventricles.

## 16 REMODELING OF THE EMBRYONIC VALVES

The last stage of the atrioventricular and outflow tract valve development involves their maturation in which the valve leaflets become slender by the remodeling of the extracellular matrix. The leaflets become organized in to three layers: (1) The atrialis of the atrioventricular valve leaflets and the ventricularis of the outflow tract valves, that is, the side of the valve facing the inflow encountering shear forces. This layer is primarily composed of radially oriented elastic fibers and provides motility of the leaflets. (2) The fibrosa, which is on the opposite side of the valve leaflet, is made up of densely organized collagen fibers and provides stiffness to the leaflets. (3) In between these two layers is the spongiosa, which is composed of proteoglycans and glycosaminoglycans. This layer allows the movement of the two flanking layers and opposes the compressive forces imposed on the valve leaflets (Hinton & Yutzey, 2011).

Most cardiac valve diseases are characterized by dysfunctional, thick and elongated valve leaflets. Histological evaluation of such valve leaflets shows that their trilaminar organization of the different layers is disrupted. A large number of extracellular matrix genes such as elastins and collagens are involved in congenital valve disease. However, upstream regulators have also been associated with valve malformation. Mutations in genes encoding extracellular matrix molecules result in remodeling of the extracellular matrix and as a consequence in dysfunctional valves. Also dysregulation of the connective tissue transcription factor Sox9, or disruption of Notch and BMP signaling results in remodeling of the extracellular matrix. If untreated this remodeling of the extracellular matrix ensues in calcifications, which will subsequently hamper valve leaflet motility and function. These features are frequently observed in human valve disease (Hinton & Yutzey, 2011).

## 17 FUTURE PERSPECTIVE

Over the last two decades the field of heart development has taken large, and sometimes bold, new steps in understanding. In spite of this new knowledge, only in a discouraging low percentage of patients with cardiac malformations, a genetic or environmental cause can be found. The hypothesis that several (genetic) events have to take place in one patient before a cardiac malformation can develop is becoming more and more likely. Therefore new insights are needed in order to understand how interactions of the now known players bring about correct cardiac development. Novel techniques identifying regions in the genome on which transcription factors act, driving their target genes, have already provided us with new knowledge on how disease could arise. To further unravel how the interplay of individual factors is orchestrated to ensure correct development of the rhythmically beating, full‐grown heart will be the aim of many researchers in this field.
